# Size of Heparin-Imprinted Nanoparticles Reflects the Matched Interactions with the Target Molecule

**DOI:** 10.3390/s19102415

**Published:** 2019-05-27

**Authors:** Yasuo Yoshimi, Daichi Oino, Hirofumi Ohira, Hitoshi Muguruma, Ewa Moczko, Sergey A. Piletsky

**Affiliations:** 1 Department of Applied Chemistry, Shibaura Institute of Technology, Tokyo 135-8548, Japan; mc16004@shibaura-it.ac.jp (D.O.); ad12022@shibaura-it.ac.jp (H.O.); 2 QOL improvement and Life Science Consortium, Shibaura Institute of Technology, Tokyo 135-8548, Japan; muguruma@sic.shibaura-it.ac.jp; 3 Department of Electronic Engineering, Shibaura Institute of Technology, Tokyo 135-8548, Japan; 4 Department of Chemistry, University of Leicester, Leicester LE1 7RH, UK; ewa@ucsc.cl (E.M.); sp523@leicester.ac.uk (S.A.P.); 5 Department of Environmental Chemistry, Catholic University of the Most Holy Conception, Concepción 4090541, Chile

**Keywords:** molecularly imprinted polymer, nanoparticles, dynamic light scattering, Zeta potential, quartz crystal microbalance

## Abstract

It has been shown that the faradic current at an electrode grafted with molecularly imprinted polymer (MIP) is sensitive to the specific target molecule used as the template. This phenomenon is applicable to sensors with very high selectivity, but the sensing mechanism is still a black box. We investigated the size sensitivity of nanoparticles of molecularly imprinted polymers (MIP-NPs) to a specific interaction for determination of the mechanism of the gate effect and its feasibility for new applications. Nanoparticles of poly(methacryloxy ethyl trimethylammonium chloride-*co*-acrylamide-*co*-methylenebisacrylamide) imprinted with heparin immobilized on glass beads were synthesized. The diameter of the MIP-NPs of heparin was increased by the presence of the heparin template but was insensitive to chondroitin sulfate C (CSC), the analogue of heparin. The high selectivity of the MIP-NPs was consistent with the selectivity of electrodes grafted with a heparin-imprinted polymer in our previous studies. The quartz crystal microbalance probes immobilizing heparin or CSC were sensitive to MIP-NPs, which indicates that the binding ability of MIP-NP does not discriminate between the template and other glycosaminoglycans. These results indicate that the size of the MIP-NP is sensitive to the matched binding with the template through the imprinted cavity.

## 1. Introduction

Molecularly imprinted polymers (MIPs) are molecular receptors composed of a synthetic polymer. A typical MIP is prepared by copolymerization of a functional monomer that has affinity to the target molecule (template) and a crosslinking monomer in the presence of the template. The extraction of the template forms a cavity in the polymer matrix capable of specific re-binding of the template or its analogues. MIPs have comparable affinity and specificity to antibodies, but they are more robust and can be prepared more economically and rapidly than antibodies. MIPs are also feasible as molecular recognition elements in biosensors [1,2,3,4,5,6,7,8,9,10,11,12,13,14,15,16]. Most of the applications of MIPs for sensors are based on the direct detection of the mass of the template bound with the MIPs (e.g., detection of the mass of the template by a quartz crystal microbalance (QCM) [2,3], detection of change in the refractive index by surface plasmon spectroscopy [4,5], or detection of the electrochemical template by stripping voltammetry [6,7]), detection by change in impedance [8], detection of the ionic template by potentiometry [9], or detection of the optical properties of the template [10].

We have been developing sensors detecting the template as an analyte indirectly by change in the faradic current of the redox marker added to the specimens at the MIP-grafted electrodes [11,12,13,14,15,16]. This method enables highly selective sensing. For example, the redox current of a phenylalanine-imprinted electrode discriminates between enantiomers even in water [13], where the binding selectivity of MIPs is usually poor. The sensing technology probably uses some morphological change in the MIP [8] on the electrode; however, the detailed mechanism is still a black box.

Recently, we developed two types of sensors for unfractionated heparin (UFH), which is a glycosaminoglycan used as an injectable anticoagulant, using poly(methacryloxyethyltrimethyl-ammonim chloride (METMAC)-*co*-acrylamide (AAm)-*co*-methylenebisacrylamide (MBAA)) imprinted with UFH. One sensor was fabricated by grafting MIP onto a flat indium–tin oxide (ITO) electrode [14,15]. The other was composed of a paste electrode fabricated by mixing oil and graphite particles grafted with MIP [16]. The faradic currents of ferrocyanide at both types of the MIP-grafted electrodes were sensitive to UFH but insensitive to chondroitin sulfate C (CSC), which is a glycosaminoglycan similar to heparin [14,15,16]. It was interesting that linear anionic polysaccharides were discriminated by the MIP, which usually recognizes the template by its 3-dimensional molecular structure. The current at the MIP-grafted flat ITO [14,15] decreased with increasing UFH concentration, whereas it increased at the paste electrode with increasing UFH concentration [16]. We have never had sufficient information to explain the reason for the discrepancy between these results from the two types of MIP electrodes. We suspect that some morphological change in the MIP may control the accessibility of the redox marker to the surface of the base electrode (ITO or graphite) responding to the specific interaction with the template heparin.

In this study, nanoparticles of poly(METMAC-*co*-MBAA-*co*-AAm) imprinted with heparin immobilized on the surface of glass beads were prepared by a modified procedure developed by one of the authors (S.P.) [17]. 

## 2. Materials and Methods

### 2.1. Chemicals

Glass beads of 50 µm in diameter (Rolloblast^®^) were purchased from Renfert Co., Ltd. (Hilzingen, Germany). *N*,*N*-Dimethylformamide (DMF) and nitric acid were purchased from Kanto Chemical Co., Ltd. (Tokyo, Japan). Benzyl diethyldithiocarbamate and 3-aminopropyltrimethoxy-silane (APTMS) were purchased from Tokyo Chemical Industry (Tokyo, Japan). Sodium salt of unfractionated heparin (UFH) (180 units/mg; from porcine intestinal mucosa: MW 15,000), acrylamide, *N*,*N*-methylenebisacrylamide (MBAA), methacrylic acid, acrylamide (AAm), dried toluene, 2-aminoethanethiol hydrochloride, and sodium chondroitin C (from shark cartilage: MW 20,000) were purchased from Wako Pure Chemical Industry Co., Ltd. (Osaka, Japan). (2-(Methacryloxy)ethyl) trimethyl ammonium chloride acrylamide (METMAC) and toluidine blue were purchased from Sigma-Aldrich, Inc. (St. Louis, MO, USA). Low-molecular-weight heparin (LMWH) was purchased as Fragmin^®^ (MW 4000–6000) from Pfizer Inc. (New York, NY, USA). 1-Ethyl-3-(3-dimethylaminopropyl)carbodiimide hydrochloride (water-soluble carbodiimide; WSC) was purchased from Dojindo Laboratories (Kumamoto, Japan).

### 2.2. Template Immobilization on Glass Beads

Heparin was covalently immobilized on the surface of the glass beads via a silane coupling agent by the following procedure. The beads were boiled in 4 M nitric acid. The washed glass beads (50 g) were soaked in a mixture of 10 g APTMS and 90 g dried toluene at 80 °C for 18 h. The treated glass beads were washed using methanol and dried at 60 °C. The glass beads were stirred in 50 mL phosphate buffer solution (pH 5.8) containing 21,600 units UFH or LMWH and WSC (0.41 mmol, 80 mg) at room temperature for 24 h. The glass beads were collected by suction filtration and rinsed sequentially with water (200 mL) and methanol (100 mL). The successful heparin-immobilization was confirmed by reddish-purple coloring in 0.01 wt% aqueous solution of toluidine blue. The beads were dried under vacuum and stored in a refrigerator.

### 2.3. Synthesis of MIP-NPs

Benzyl diethyldithiocarbamate (0.15 g, photoinitiator of radical polymerization), MBAA (0.5 g, crosslinker), AAm (0.50 g, crosslinking regulator), and METMAC (0.45 g, functional monomer) were dissolved in a mixed solvent of *N*, *N*-dimethylformamide (18 mL) and distilled water (6 mL) to prepare the prepolymer solution. The UFH- or LMWH-immobilized glass beads (5 g) and the prepolymer solution were dissolved in a quartz crystal test tube (25 mm inner diameter) through which nitrogen was bubbled for 20 min in order to purge oxygen. The mixture was irradiated for 15 min with a Xenon lamp LC5 (peak wavelength 365 nm, 4.5 W/cm^2^, Hamamatsu Photonics Co., Ltd., Hamamatsu, Japan) from the bottom of the tube through an optical fiber to promote radical polymerization. The glass beads were washed successively using DMF (75 mL) and distilled water (25 mL) by suction glass filtration. The glass beads were flushed at 60 °C with 1 M sodium chloride solution (100 mL) to remove polymers weakly adsorbed on the beads. The glass beads were transferred into a 300 mL beaker filled with 100 mL of 1 M NaCl solution.

The glass beads were stirred in the brine at 80 °C for 1 h using a heating magnetic stirrer to dissociate the MIP-NPs strongly bound to the template on the beads. The MIP-NP dispersion was collected by filtration with a 60 mL SUPELCO tube (Sigma-Aldrich, St. Louis, MO, USA) with a filter of 20 µm pore diameter by air pressure. The dispersion was packed in a Visking Tube (24.5 mm diameter, Viskase Inc., Willowbrook, IL, USA) for dialysis with distilled water until the electroconductivity of the dispersion became equal to that of distilled water. NaCl was added to the dialyzed dispersion to a concentration of 0.15 M (the same as physiological saline). (We attempted to prepare non-imprinted polymer nanoparticles as a reference by the same procedure except by using acid-washed glass beads without the heparin coating. However, no polymer particles were obtained. This is probably due to the interaction between the synthesized polymer and the surface of the unmodified glass beads being too weak.) The diameter of the MIP-NPs was measured by dynamic light scattering (DLS) (DelsaMax Pro, Beckman Coulter, Brea, CA, USA). The relationship between the radius of the MIP-NPs and the concentrations of sodium glycosaminoglycans (UHF, LMWH and CSC) was compared. The zeta potential of the MIP-NPs was measured by DelsaMax (electric field frequency was 10 Hz, voltage amplitude was 2.5 V). 

### 2.4. Evaluation of the Binding Ability of MIP-NPs with Glycosaminoglycans of Quartz Crystal Microbalance (QCM)

Heparin or CSC was covalently immobilized on a gold layer of a 5 MHz QCM sensor (QSX301, Biolin Scientific, Gothenburg, Sweden) as follows. The QCM sensor probes were soaked in 1 mM ethanol solution of 3-aminoethanethiol hydrochloride for 24 h to aminate the surface of the gold electrode of the sensor. The sensor was washed using ethanol and dried using a stream of nitrogen gas. The surface-aminated QCM sensor was soaked in 50 mL phosphate buffer solution (pH 5.8) containing 120 mg UFH or CSC and 80 mg (0.41 mmol) WSC at room temperature for 24 h. The UFH- or CSC-coated QCM sensor was washed using distilled water. The treated QCM or nontreated QCM was installed in the flow cell of the QCM measurement system (QSense Explorer, Biolin Scientific, Gothenburg, Sweden). Initially, physiological saline was flowed through the cell with a flow rate of 1 mL/min until the frequency was stabilized. Next, the dispersion of MIP-NP templated by UFH (UFH-MIP-NP) was flowed through the same line. The natural oscillation frequency of the QCM changed due to the binding between MIP-NP and UFH or CSC immobilized on QCM.

## 3. Results

### 3.1. Sensitivity of the Size of MIP-NPs to the Template or Analogue

**Figure 1:**
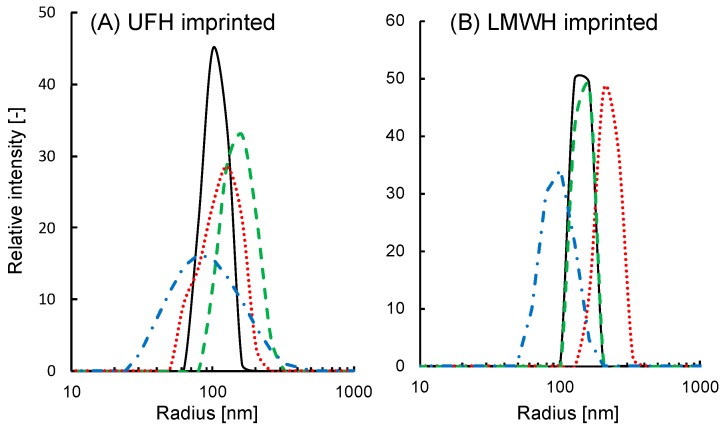
A comparison of the radius distributions of (**A**) the polymer nanoparticles imprinted by unfractionaled heparin (UFH-MIP-NP) and (**B**) those imprinted with low molecular weight heparin (LMWH-MIP-NP) in the absence of glycosaminoglycan (black solid lines) and in the presence of UFH (green dashed lines), LMWH (red dotted lines) or chondroitin sulfate C (CSC) (blue dot-dashed lines). The concentration of the glycosaminoglycan is 1 units/mL with UFH-MIP-NP and 5 units/mL with LMWH-imprinted nanoparticles.

**Figure 2:**
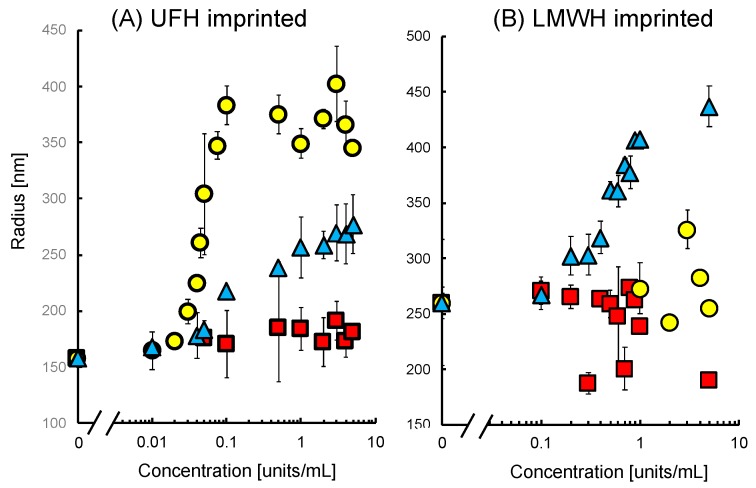
The relationship between the radii of (**A**) UFH-MIP-NP and (**B**) LMWH-MIP-NP and the concentrations of UFH (yellow circles), LMWH (blue triangles), and chondroitin sulfate C (CSC) (red squares).

The effects of UFH, LMWH, and CSC on the radius of the UFH-imprinted NP (UFH-MIP-NP) or LMWH-imprinted NP (LMWH-MIP-NP) are shown in Figure 1. The average radius of the imprinted nanoparticles as a function of the concentration of glycosaminoglycans is shown in Figure 2. The average radii of both types of MIP-NPs were insensitive to CSC (red squares). However, the addition of CSC broadened the distribution of the radii of the MIP-NPs. These results suggest that both types of MIP-NPs interact with CSC, where this interaction increased the size of a portion of the particles but decreased that of another portion; thus, it does not lead to noticeable change in the average diameter. The radius of UFH-MIP-NPs increased up to 2.5 times in response to addition of the template UFH but was much less influenced by LMWH. 

The radius of the LMWH-MIP-NPs increased by addition of LMWH, but it was not influenced by the addition of UFH. These observations indicate that the increase in the size of MIP-NPs resulted from the specific interaction of the imprinted cavity and the template. The UFH-MIP-NP has such a large imprinted cavity that it could partially accommodate LMWH; thus, its effect on the diameter of these particles was small. In contrast, the small cavity in LMWH-MIP-NPs could not form a stable interaction with UFH; thus, it had no noticeable effect on the size of the LMWH-MIP-NPs.

### 3.2. Sensitivity of the QCM Sensor Coated with the Template or Analogue to the MIP-NPs

**Figure 3:**
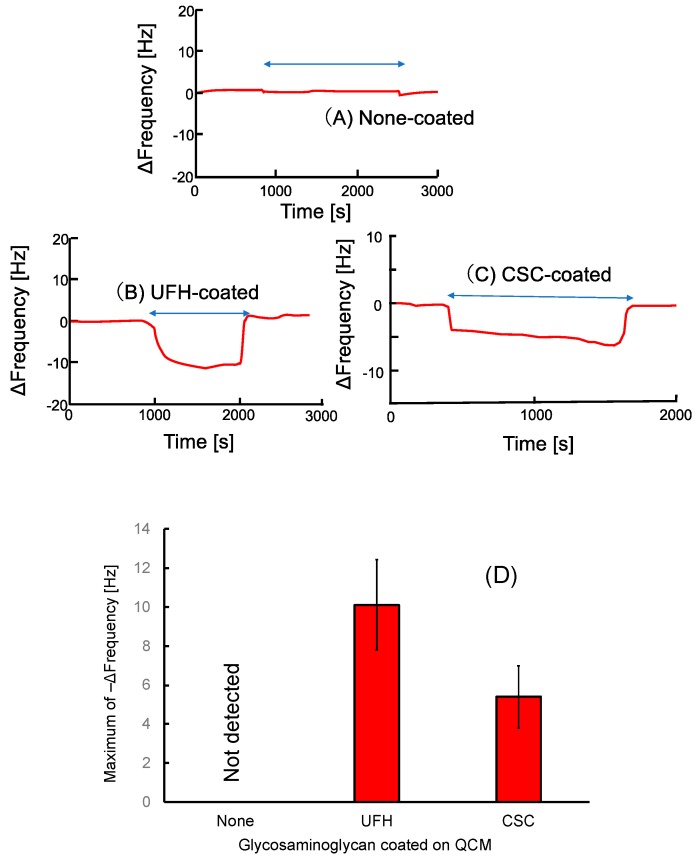
Time courses of the frequencies of the quartz crystal microbalance (QCM) sensors coated with (**A**) no coating, (**B**) UFH, or (**C**) CSC by exposure to UFH-imprinted nanoparticles during the time indicated by the two-directional arrows. (**D**) The maximum change in the frequency during UFH-MIP-NP exposure at the QCM.

Time courses of the natural oscillation frequencies of the QCM sensors are shown in Figure 3. The frequency of untreated QCM was insensitive to exposure to the UFH-MIP-NPs (Figure 3A). This indicates that adhesion of MIP-NPs at the surface of the QCM due to aggregation of the particle or affinity with the gold surface did not occur. However, the exposure to MIP-NPs decreased the frequency of the QCM coated with UFH (Figure 3B) or CSC (Figure 3C). The frequencies of the QCM in physiological saline before and after exposure to the MIP-NPs were almost the same. This indicates that MIP-NPs templated with UFH bind reversibly with CSC as well as with UFH. The change in the frequency during UFH-MIP-NP exposure at the UFH-coated sensor was larger than that at the LMWH-coated one, as shown in Figure 3D. The difference in the change does not necessarily indicate that the UFH-MIP-NPs bind with UFH better than CSC because UFH-MIP-NPs would also swell when responding to UFH on the QCM to generate the larger frequency change. However, the results clearly show that the UFH-MIP-NPs do not bind only with UFH but also with CSC.

### 3.3. Sensitivity of the Zeta Potential of the MIP-NPs to the Template or the Analogue 

**Figure 4:**
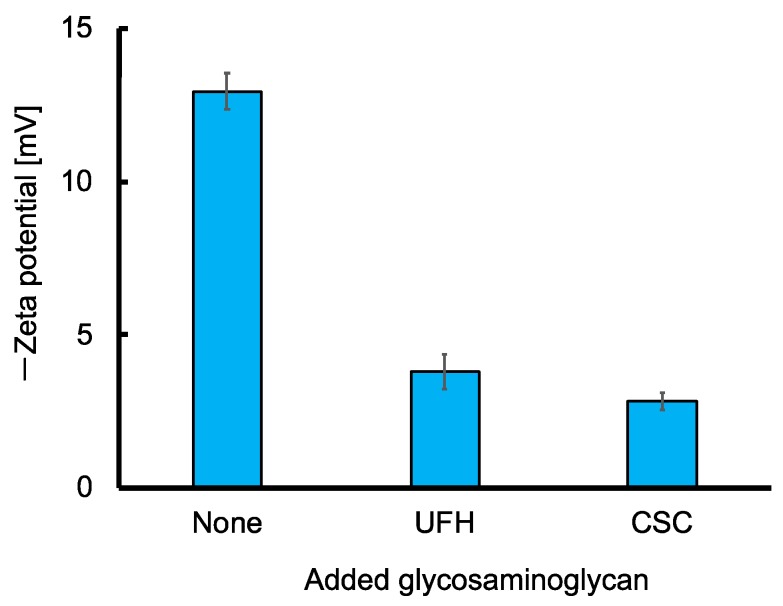
The effect of the addition of 15 units/mL glycosaminoglycans (UFH or CSC) on the zeta potential of UFH-MIP-NPs.

The zeta potential of UFH-MIP-NPs was −12.9 ± 0.6 mV. The negative potential indicates that the surface (inside the slipping plane [18]) of the UFH-MIP-NP is negatively charged, although it contains cationic trimethylammonium groups originating from METMAC. It is likely that the trimethylammonium groups exist in the bulk of the UFH-MIP-NP, but the surface of the particle is rich in the chloride counter anion. The potential shifted positively by the addition of 15 units/mL of UFH or CSC, as shown in Figure 4. The declines in the negative zeta potential by both UFH and CSC indicate that both glycosaminoglycans were absorbed in the UFH-MIP-NP by binding with trimethylammonium groups through electrostatic interactions, thus liberating anions on the surface of the particles. Therefore, the zeta potential results support the idea that the UFH-MIP-NP binds with CSC as well as UFH. Nanoparticles of UFH-imprinted poly(METMAC-*co*-MBAA-*co*-AAm) bind with both the UFH-template and CSC, which is an analogue of the template, but the size was enhanced only by the UFH template and LMWH, which is a fragment of the template. However, the broadening of the radius distribution by CSC indicates an interaction between the CSC and the MIP-NPs, which is consistent with the results of the QCM. 

## 4. Discussion

Nanoparticles of UFH-imprinted poly(METMAC-*co*-MBAA-*co*-AAm) bind with both the UFH template and CSC, which is an analogue of the template, but the size was enhanced only by the UFH template and LMWH, which is a fragment of the template. However, the broadening of the radius distribution by CSC indicates an interaction between the CSC and the MIP-NPs, which is consistent with the results of the CSC-immobilized QCM. 

**Figure 5:**
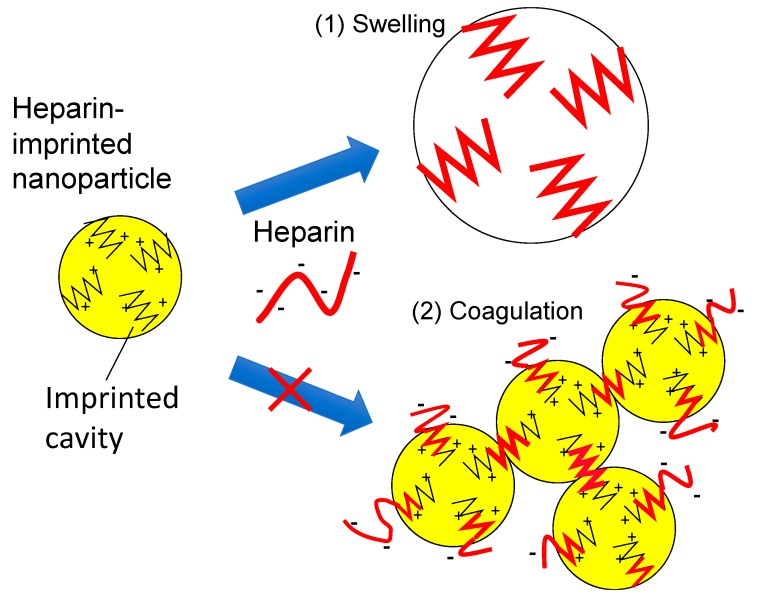
Scheme of hypothetical mechanisms for the size increase of the heparin-imprinted nanoparticles by specific interaction with the template.

The mechanism of size enhancement of the nanoparticles is attributed to either (1) swelling of the polymer matrix or (2) coagulation of the particles by crosslinking via the template binding with two or more particles (Figure 5). Nanoparticle coagulation resulting from crosslinking by the template is unlikely due to the following four reasons.

(a) The UFH-imprinted MIP-NP also binds with CSC as indicated by QCM. Thus, CSC would also have crosslinked particles based on the coagulation assumption. Therefore, the hypothesis is inconsistent with the result that the radius of the particle was insensitive to CSC.
(b) Figure 2 shows that the size increase of UFH-imprinted MIP-NPs by UFH was saturated. If the increase resulted from coagulation, it would not be saturated.
(c) The long-chain UFH is more advantageous than short-chain LMWH for crosslinking the MIP nanoparticles. However, the size of the UFH-imprinted nanoparticles increased in the presence of LMWH, whereas the size of LMWH-MIP-NPs was insensitive to UFH.
(d) If bound UFH existed on the surface of the UFH-MIP-NPs, the zeta potential would have shifted negatively. However, the result was the opposite. It is likely that UFH or CSC was absorbed in the bulk of the particles, preventing the glycosaminoglycans from crosslinking the nanoparticles.

These inconsistencies reveal that it is unlikely that radius enhancement occurred by coagulation resulting from crosslinking by heparin bound with the MIP-NPs. 

**Figure 6:**
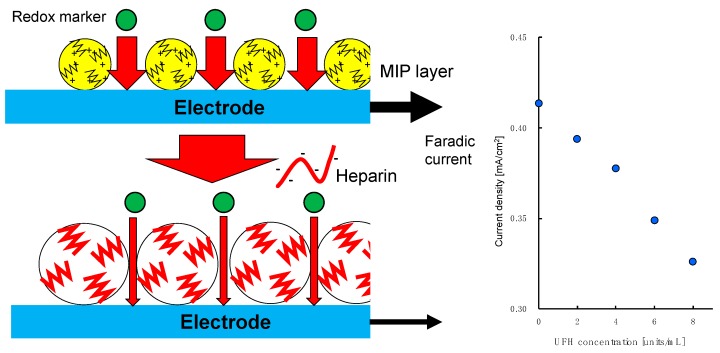
Scheme of the possible mechanism of heparin sensing with an indium–tin oxide electrode grafted with UHF-MIP where the faradic current decreased with increased UFH concentration as shown on the right-hand side [15].

The swelling of the polymer matrix in MIP-NPs by specific interactions can be explained using the results of our previous works [14,15,16]. We have been developing a heparin sensor by grafting heparin onto the surface of electrodes. Since the present and previous experiments with MIP-NPs were both performed in physiological saline (0.15 M NaCl), the results can be compared. An electrode grafted with UFH-imprinted poly(METMAC-*co*-MBAA-*co*-AAm) generated a faradic current that was sensitive to UFH but insensitive to CSC. However, the faradic current of ferrocyanide at the MIP-grafted flat electrode of indium–tin oxide was decreased by heparin. We can explain the mechanism by recognizing the MIP-grafted layer as an assembly of small particles of MIP-NPs. The nanoparticles would be swelled by the specific interaction with the template, and the pathway for diffusion of the redox solute in the MIP layer would be decreased as shown in Figure 6. (We have termed this phenomenon the “gate effect” [11,12,13,14,19].) This is why the current at the UFH-MIP grafted flat electrode was decreased by the UFH [14,15]. This hypothesis is also consistent with the result that the size of the UFH-MIP-NPs and the faradic current at the MIP-grafted ITO were less sensitive to LMWH [15] than to UFH and were insensitive to CSC. 

**Figure 7:**
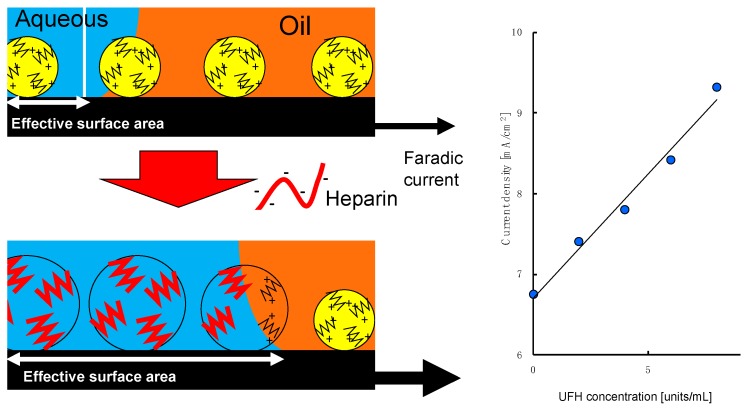
Scheme of the possible mechanism of heparin sensing with a paste electrode produced by mixing oil and graphite particles grafted with UFH-MIP, where the faradic current increased with the UFH concentration as shown on the right-hand side [16].

In contrast, the faradic current of ferrocyanide at a paste electrode made by mixing silicone oil and UFH-MIP-grafted graphite particles was increased by increasing heparin concentration [16]. The ferrocyanide anion and heparin are both hydrophilic; thus, they cannot approach the surface of the electrode across the oil used for binding the MIP-grafted graphite particles to form the paste electrode. The heparin binding and redox reaction of ferrocyanide would occur at the limited surface region of the MIP-grafted electrode that is in direct contact with the aqueous phase. The expansion of the MIP layer by heparin binding would increase the hydrophilicity of the surface of the grafted particles and push the oils in the bulk of the paste electrode, as shown in Figure 7. These phenomena contribute to an increase in the effective surface area for the electrochemical reaction and increase the redox current with increasing heparin concentration. The redox current at these UFH-MIP-grafted electrodes was insensitive to CSC and less sensitive to LMWH than to UFH in physiological saline. Our previous results are therefore consistent with the present results. 

We developed another variation of a heparin sensor by grafting MIPs of UFH onto the surface of a QCM sensor to detect the mass of bound heparin directly [20]. The frequency decreased upon exposure to both CSC and UFH. This is consistent with the present results indicating that UFH-MIP-NPs bound with both UFH and CSC on the QCM sensor. Regarding the change in the QCM frequency shown in Figure 3, a larger number of MIP-NPs seem to bind with the UFH on the QCM than with the CSC. However, the UFH-MIP-NPs bound with UFH on QCM are also thought to expand; thus, we cannot conclude that the MIP-NP binds with UFH better than with CSC.

There is an inconsistency between the dynamic ranges of the UFH concentration for the expansion of MIP-NPs (0.03–0.1 units/mL) and that for the current change at the MIP electrode (1–20 units/mL) [15]. This is likely due to the accessibility limitation of the UFH to and through the MIP layer fixed on the electrode.

We have yet to obtain definitive information to explain the mechanism of how the matched binding with the template expands the matrix of the MIP while the mismatched binding with an analogue of the template does not. Previously, one of the authors (S.P.) and colleagues discussed template-induced swelling as a result of entropy-driven binding phenomena observed for natural and synthetic receptors [21]. Basically, when the template is extracted from the synthesized MIP, polymer chains of the MIP tend to shrink due to hydrophobic interaction between adjacent polymer chains in the MIP. Rebinding with MIP template bonds destroys the network of the hydrophobic interaction [22], thus allowing water molecules to be re-absorbed in the MIP matrix and causing the polymer swelling. Watanabe et al. similarly discussed their result that an adrenaline- or noradrenaline-imprinted hydrogel swelled by specific interaction with its template [23]. If the MIP-NP was composed of more hydrophobic materials (e.g., ethyleneglycol dimethacrylate), the hydrophobic interaction might have been stronger; thus, the hydrophobic MIP-NPs would be too rigid to indicate swelling by template-specific interaction. However, if the material were more hydrophilic, the swelling might have been saturated without the template-specific interaction or too flexible to discriminate between the matched and the mismatched bindings [13,24,25]. The exploration of this mechanism would be valuable for the development of more highly selective sensors using morphological change in MIPs by matching binding with the template rather than sensors detecting templates bound with MIPs directly. 

## 5. Conclusions

A nanoparticle of poly(METMAC-*co*-MBAA-*co*-AAm) imprinted by a heparin template swells by interaction with the template but is not swelled by CSC, which is the analogue of heparin. However, the particle can bind with both heparin and CSC. The findings demonstrate that molecularly imprinted polymer nanoparticle swelling is sensitive to the matched binding with the template but insensitive to mismatched binding with the analogue. This idea is consistent with the results of our previous works where amperometric electrodes grafted with heparin-imprinted polymer could discriminate between heparin and CSC but a QCM grafted with the MIP is sensitive to both. The application of the swelling phenomena of the MIPs responding to matched binding would be advantageous for sensing the target molecule used as the template of the MIP with high selectivity.
