# Erratum: Yoshimi, Y., *et al.* Size of Heparin-Imprinted Nanoparticles Reflects the Matched Interactions with the Target Molecule. *Sensors* 2019, *19*, 2415

**DOI:** 10.3390/s19173646

**Published:** 2019-08-21

**Authors:** Yasuo Yoshimi, Daichi Oino, Hirofumi Ohira, Hitoshi Muguruma, Ewa Moczko, Sergey A. Piletsky

**Affiliations:** 1 Department of Applied Chemistry, Shibaura Institute of Technology, Tokyo 135-8548, Japan; 2 QOL improvement and Life Science Consortium, Shibaura Institute of Technology, Tokyo 135-8548, Japan; 3 Department of Electronic Engineering, Shibaura Institute of Technology, Tokyo 135-8548, Japan; 4 Department of Chemistry, University of Leicester, Leicester LE1 7RH, UK; 5 Departamento de Química Ambiental, Facultad de Ciencias, Universidad Católica de la Santísima Concepción, Concepción 4090541, Chile

The authors wish to make the following erratum to this paper [1]:

The affiliation 5 of co-author Ewa Moczko should be corrected into:

“Departamento de Química Ambiental, Facultad de Ciencias, Universidad Católica de la Santísima Concepción, Concepción 4090541, Chile”.

The authors would like to apologize for any inconvenience caused to the readers by these changes.
